# The -1364A/C Aquaporin 5 Gene Promoter Polymorphism Is Not Associated with Menière's Disease

**DOI:** 10.5402/2012/706896

**Published:** 2012-10-16

**Authors:** Diana Arweiler-Harbeck, Freschta Saidi, Stephan Lang, Jürgen Peters, Winfried Siffert, Michael Adamzik

**Affiliations:** ^1^Klinik für Hals-Nasen-Ohrenheilkunde, University of Duisburg-Essen, 45122 Essen, Germany; ^2^Klinik für Anästhesiologie und Intensivmedizin, University of Duisburg-Essen and Universitätsklinikum Essen, 45122 Essen, Germany; ^3^Institut für Pharmakogenetik, University of Duisburg-Essen, 45122 Essen, Germany

## Abstract

*Objective*. Aquaporin 5 plays an important role in maintaining inner ear water and fluid homeostasis. Since the aquaporin (AQP) 5 promoter-1364A/C polymorphism is associated with altered AQP5 expression and this could impact upon key mechanisms of Menière's disease, we tested the hypothesis that genotypes of the AQP5 promoter-1364A/C polymorphism are associated with the incidences of Menière's disease (MD), familial Menière's disease (FMD), or endolymphatic hydrops (EH). *Methods*. With approval of the local ethics committee, DNA of 102 patients (39 with MD, 54 with FMD, and 9 with EH) and of 292-matched Caucasian controls was isolated from blood samples and genotyped for the *AQP 5* promoter-1364A/C polymorphism. The *χ* ^2^-test was applied to compare genotype distributions and allele frequencies between patients and controls. *Results*. Overall, genotype frequencies were not different between controls (AA 69%, AC 30%, CC 1%) and patients with MD AA: 65.7% (23 MD, 37 FMD, and 8 EH); AC: 23.5% (12 MD, 11 FMD, and 1 EH); CC: 3.9% (1 MD, 3 FMD, and 0 EH). However, subgroup analysis revealed the CC genotype to be more frequent in patients with FMD (5.9%) than in healthy controls (1%) (*P* = 0.042). *Conclusions*. Overall, genotypes of the -1364A/C *AQP5* gene polymorphism are not associated with a significant increased risk for Menière's disease.

## 1. Introduction

Although the pathogenesis of Menière's disease remains unknown it is likely to be multifactorial. One of these factors could be a genetic predisposition. A candidate is the gene encoding aquaporin 5 (*AQP5*) as aquaporins play an important role in maintaining inner ear fluid homeostasis. Furthermore, *AQP5* is preferentially localized within the external sulcus cells and the spiral prominence of the cochlea implying a physiological role of AQP5 in auditory function [1, 2].

Following sequencing of the *AQP5* promoter region of 50 healthy white Caucasians we have described a novel, functional, and common single nucleotide (-1364A/C) polymorphism [3]. Substitution of C for A at position -1364 was associated with increased binding of transcription factors, as shown for nuclear extracts from HeLa cells, but reduced transcriptional activation of the *AQP5* gene in response to serum and camp [3]. This latter finding is of special interest since stimulation of the vasopressin V2-receptor by arginine-vasopressin (ADH) results in increased AQP5 mRNA concentrations and increased translocation of AQP5 to the plasma membrane [3]. This is mediated *via* an increase in cAMP concentration and subsequent activation of the protein kinase A pathway. Thus, the C-allele may facilitate the binding of an inhibitory transcription factor. These results were corroborated by showing that the C-allele was associated with decreased AQP5 mRNA transcripts in human right atrial muscle and decreased AQP protein expression in red cell membranes [3]. Furthermore, the C-allele was associated with suppression of the renin-angiotensin-aldosterone system (RAAS) in response to a high salt diet, while the RAAS suppression in individuals carrying the AA genotype was significantly blunted. Thus, there are good reasons to hypothesize that the *AQP5 *promoter polymorphism could impact upon key mechanisms of MD.

Accordingly, we tested the hypothesis that genotypes of the -1364A/C *AQP5* promoter polymorphism are associated with an increased risk for MD, FMD, or EH.

## 2. Material and Methods

After approval by the local ethics committee and written informed consent data and blood samples from 102 caucasian patients with Menière's disease (grade 1–4 according to AAO-HNS guidelines [4]) were included into the study cohort (62 females, 40 males; mean age: 59.1 years ±14.3 (max 96, min 25), see Table 1)). The DNA of 102 patients, 39 with Menière's disease (MD), 54 with familial Menière's disease (FMD), and 9 with endolymphatic hydrops (EH) was genotyped for the *AQP5* promoter-1364A/C polymorphism. Familial Menière's disease (FMD) was assumed when two or more family members were affected by Menière's disease. Endolymphatic hydrops (EH) was defined as fluctuating low-tone hearing loss with no signs of vestibular affection. Other pathologies of the inner ear were excluded by magnetic resonance imaging. 

All patients were recruited at the local Department of Otorhinolaryngology, University Hospital Essen. The group consisted of patients referred to the department for diagnosis and/or therapy of Menière's disease or endolymphatic hydrops by smaller departments or local ENT colleagues. As patients were usually referred to the department when standard conservative treatment was not successful, the groups of patients with grade 2 (*n* = 27) and grade 3 (*n* = 53) disease are the largest of our cohorts (see Table 2(b)). The details of patients with familial Menière's disease have already been reported [5]. 

### 2.1. Control Group

The control cohort consisted of 292 randomly chosen healthy white, caucasian blood donors (age: 56.7 years ± 4.1) of either gender (110 female, 182 male) who were recruited at the local Department for Transfusion Medicine, University Hospital Essen. The details of this cohort have been reported previously [6, 7].

### 2.2. Determination of Genotypes

DNA was extracted from whole blood using the QIAamp kit (Qiagen, Hilden, Germany). For genotyping the AQP5 promotor-1364 A/C polymorphism by Pyrosequencing, PCR was performed with the forward primer AQP5-SE 5′-GAAACTGCAGGATGAGAGAAAT-3′, and the biotinylated reverse primer AQP5-AS 5′-TCTCTGTTCTCCACCTCTCCA-3′ resulting in a 120 nt fragment. After denaturation at 94°C, 40 cycles of DNA amplification were done using Taq PCR Mastermix (Eppendorf, Hamburg, Germany) at 94°C for 40 s, 53°C for 40 s, and 72°C for 40 s. The biotinylated strand was captured on streptavidin sepharose beads and annealed with a sequencing primer AQP5-Seq 5′-CAGAGAGACTAAGACAGCA-3′. Pyrosequencing was performed using PSQ HS 96 Gold SNP Reagents and the PSQ HS 96 (Biotage, Uppsala, Schweden).

### 2.3. Statistical Analyses

All statistical analyses were done using SPSS 11.0 (SPSS, Chicago, IL, USA). Continuous variables are given as means ± SD, as indicated. The Hardy-Weinberg equilibrium was tested by a goodness-of-fit *χ* ^2^-test. The *χ* ^2^-test was applied to compare genotype distributions and allele frequencies between patients and controls. Differences were regarded statistically significant with an alpha error *P* of less than 0.05. 

## 3. Results

Genotype frequencies were not different between controls (AA: 69%, AC: 30%, and CC: 1%) and patients with MD AA: 65.7% (23 MD, 37 FMD, and 8 ED); AC = 23.5% (12 MD, 11 FMD, and 1 ED); CC = 3.9% (1 MD, 3 FMD) (Table 2(a)). Genotype distribution in patients was compatible with the Hardy-Weinberg equilibrium. 

Subgroup analysis, however, revealed the CC genotype to be more frequent in patients with FMD than in controls (*P* = 0.042). However, neither the A-allele nor the C-allele was associated with FMD or MD. 

Distribution of comorbidities varied among the subgroups (Table 1). Allergies, metabolic disorders (diabetes mellitus, impairment of cholesterol metabolism), hypertension, and depression were significantly more frequent in the group of patients with MD than in patients with FMD. However, there was no significant association between these disorders and specific genotypes of the *AQP5* promoter polymorphism in these patients.

Additionally, severity of MD was not associated with the *AQP5* promoter-1364A/C polymorphism, as depicted in Table 2(b). However, this calculation has to be interpreted carefully as the different grades are only a “snapshot” in the course of disease in patients presenting for diagnosis or therapy. Anyhow, the individual course of MD is variable and cannot be foreseen.

## 4. Discussion 

The present investigation suggests for the first time that genotypes of the -1364A/C *AQP5* promoter polymorphism are neither associated with the incidence nor the severity of MD, FMD, or EH. Although the CC genotype was more frequent in patients with FMD than in controls (*P* = 0.042), we believe that this association is fortuitous because neither the A-allele nor the C-allele was associated with FMD or MD. 

Comorbidities such as allergies, metabolic disorders, hypertension, and depression were more frequent in patients with MD than in those with FMD. With regard to the relatively small number of patients in each group these results have to be interpreted carefully. However, it is well known that depression and allergies might act as cofactors or so-called “triggers” evoking Menière attacks [8, 9]. 

The preferential presence of AQP5 within the external sulcus cells and the spiral prominence of the cochlea implies a physiological role of AQP5 in auditory cell function and inner ear water homeostasis [1, 2] and the -1364A/C aquaporin 5 gene promoter polymorphism impacts upon AQP5 expression in different cohorts [3]. Nevertheless, the -1364A/C aquaporin 5 gene promoter polymorphism was not associated with the incidence of MD, FMD, or EH. We speculate, therefore, that AQP5 expression in the apical turns of the cochlea only may play a limited role in inner ear fluid homeostasis in patients with Menière's disease. 

This speculation is supported by observations of Klar et al. who defined a new candidate region for an increased risk for FMD on chromosome 12 [10]. Although this region on chromosome 12p12 is close to the AQP 2, 5, and 6 loci, there was no overlapping or linkage disequilibrium between these loci. Furthermore, Merves et al. [11] reported normal hearing in AQP5 knockout mice and suggested redundant or alternative mechanisms for regulation of water homeostasis in the inner ear. 

However, according to the findings of Hirt and coworkers [12], there should be further attention paid to the special differential localization and role of AQP5 and AQP 4 in the outer sulcus cells, as it suggests a possible water shunt in the perilymph—endolymph barrier which could have an impact on dysfunctional water regulation such as endolymphatic hydrops or sensorinureal hearing loss. 

Limitations of this report should be mentioned. The study population was relatively small and it was hardly possible to build matched pairs as far as comorbidities and age are concerned. Different selection criteria of blood donors and an unrecognized selection bias, inherent to many genetic association studies, cannot be excluded, too.

Factors predicting the onset or severity of MD and EH are hardly known. However, the low incidence of MD, FMD, and EH in the general population suggests an interaction of extrinsic (e.g., allergies, inflammation, metabolic and endocrinological disorders, trauma, and migraine), genetic, and developmental intrinsic factors. This investigation represents a further step towards identifying candidate genes which could impact on the onset or severity of MD and EH. However, in this survey on Caucasians neither comorbidities nor the AQP5 polymorphism was associated with the incidence or severity of MD, FMD, or EH.

## Figures and Tables

**Table 1:** Demographics of patients with Meniere's disease (*n* = 102) and of controls (*n* = 292).

	Patients		Controls	
Age (years)	59.1 ± 14.3		56.7 ± 4.1	
Sex, male/female *n* (%)	62 (60.8)/40 (39.2)		110 (37.7)/182 (62.3)	

Grade of disease according to AAO-HNS (*n* = 93)	MD (*n* = 39)	FMD (*n* = 54)		

1	3	5		
2	14	13		
3	22	31		
4	0	5		

Endolymphatic hydrops	9			

Comorbidities	MD	FMD	EH	*P* value

Thyroid disorders	7	8	3	0.1
Allergies	16	9	3	**0.048**
Migraine	6	12	2	0.627
Metabolic disorders	13	2	1	**0.022**
Hypertension	16	9	3	**0.048**
Depression	11	5	0	**0.024**

Age is presented as: means ± SD (standard deviation); statistical significance (*P* < 0.05) of frequency of comorbidities within the subgroups (MD, FMD, and EH) is indicated by the *P* value.

**Table:** (a)

Genotypes/alleles/entities	AA	AC	CC	*P* value	A	C	*P* value
	RowSpanEmpty	*n* (%)	*n* (%)		*n* (%)	RowSpanEmpty		*n* (%)	*n* (%)
EH	8	1	0	0.352	17	1	0.334
	(88.9)	(11.1)	0		(94.4)	(5.6)	

MD	23	12	1	0.391	58	14	0.498
	(63.9)	(33.3)	(2.8)		(80.6)	(19.4)	

FMD	37	11	3	0.042*	85	17	0.884
	(72.5)	(21.6)	(5.9)		(82.7)	(17.3)	

Controls	202	87	3		491	93	
	(69.9)	(30)	(1)		84.1	(15.9)	

*P* values refer to statistical significance (*P* < 0.05) of genotype frequencies within the particular groups of EH, MD, and FMD. *P* value of CC in the FMD group (0.042) is considered as statistically significant.

**Table:** (b)

Genotypes/stadium	AA	AC	CC	All
EH	8	1	0	9
Grade 1	7	1	0	8
Grade 2	20	6	0	26
Grade 3	30	15	3	45
grade 4	3	1	1	5
